# Association between a frailty index derived from laboratory tests and clinical outcomes in critical care patients with asthma: a retrospective study based on the MIMIC-IV database

**DOI:** 10.3389/fmed.2025.1539531

**Published:** 2025-09-18

**Authors:** Jianmin Qu, Tingting Wang, Zhe Li, Yi Yu

**Affiliations:** ^1^Department of Intensive Care Unit, Tongxiang First People's Hospital, Tongxiang, Zhejiang, China; ^2^Department of Intensive Care Unit, The Second People's Hospital of Liaocheng, Linqing, Shandong, China; ^3^Department of Intensive Care Medicine, Juancheng County People's Hospital, Heze, Shandong, China; ^4^Department of Critical Care Medicine, The Second Affiliated Hospital of Guangzhou University of Chinese Medicine, Guangzhou, Guangdong Province, China

**Keywords:** FI-LAB, asthma, mortality, ICU, MIMIC-IV

## Abstract

**Background:**

In this study, we aimed to explore the association between elevated Frailty Index laboratory-derived (FI-Lab) levels and the prognosis in critically ill patients with asthma to advance clinical management strategies and optimize patient outcomes.

**Methods:**

A retrospective analysis was performed on intensive care unit (ICU) patients diagnosed with asthma identified from the Medical Information Mart for Intensive Care (MIMIC-IV, version 3.0) database. Demographic characteristics, clinical parameters, and FI-Lab values were collected for analysis. The prognostic factors were evaluated using multivariate Cox regression models.

**Results:**

Our findings revealed a strong association between elevated FI-Lab levels and adverse clinical outcomes in ICU patients with asthma. Among the 2,339 patients, those presenting with higher FI-Lab scores at admission exhibited a significantly increased risk of complications, including prolonged ICU stays and higher mortality rates. Specifically, a 0.1-unit increase in the FI-Lab score was associated with a 1.32-fold increased risk of 28-day mortality [hazard ratio (HR) = 1.32, 95% confidence interval (CI): 1.17–1.50, *p* < 0.001], 1.25-fold increased risk of ICU mortality (HR = 1.25, 95% CI: 1.05–1.47, *p* = 0.011), and 1.33-fold increased risk of 90-day mortality (HR = 1.33, 95% CI: 1.20–1.48, *p* < 0.001). Additionally, when the FI-Lab scores were analyzed as categorical variables, significant associations with the 28-day, ICU, and 90-day mortality rates remained consistent across all models.

**Conclusions:**

Elevated FI-Lab levels are a strong prognostic factor in ICU patients with asthma. Incorporating the FI-Lab into clinical evaluations may provide a new scientific basis for assessing the prognosis of severe asthma and potentially improving patient outcomes. Randomized controlled trials are required to validate our findings.

## Background

Asthma is a prevalent respiratory disease affecting more than 300 million individuals globally (1). Despite advances in treatment, asthma control remains suboptimal, with nearly half of adult patients experiencing acute exacerbations annually (2, 3). In the United States alone, approximately 25,000–50,000 patients with asthma require admission to the intensive care unit (ICU) for treatment each year (4). Therefore, early identification of high-risk patients and timely intervention are essential to reduce mortality rates in this vulnerable population.

The prognostic assessment of critically ill patients with asthma remains a significant clinical and scientific challenge. Traditional prediction tools, such as the Acute Physiology and Chronic Health Evaluation and the Systemic Inflammatory Response Syndrome score, have demonstrated limitations in predicting short- and long-term mortality, particularly in individualized prognostic contexts (5, 6). Although pediatric asthma risk scores such as the asthma predictive index and clinical respiratory score (CRS) are commonly used to assess asthma conditions (7, 8), they do not fully capture the systemic condition of critically ill patients and, thus, exhibit notable shortcomings in assessing severity and prognosis. Most published asthma severity scores were developed for clinical trials and may not be universally applicable to diverse patient populations in clinical settings (9, 10). For the CRS to be considered clinically valid, it must exhibit reliability, user-friendliness, and responsiveness. Some components of currently utilized asthma scores may lack validation, thereby limiting their value in guiding medical decision-making. Thus, an urgent need exists for identifying new predictive markers that can accurately predict patient prognosis.

Frailty is a biological syndrome characterized by reduced physiological reserves and impaired function of multiple organ systems, making individuals more vulnerable to adverse health outcomes (11). The latest update in 2023 recognizes that frailty can be defined as and is pertinent to individuals with various chronic respiratory conditions, including asthma (12). Individuals with asthma are more likely to exhibit the characteristics associated with frailty, including decreased muscle strength, endurance, and immune function (13). The prevalence and association of frailty with clinical outcomes among patients with asthma indicate that frailty exacerbates disease symptoms and healthcare burden (14). Furthermore, frailty is associated with a higher mortality rate in ICU patients (15). Traditional frailty assessment methods often require face-to-face evaluations and special tools such as grip strength measurements, which can be challenging in clinical practice (16). Howlett et al. (17) introduced a composite index derived from objective laboratory tests and vital signs (FI-Lab) that can be readily calculated during routine clinical practice. This index has demonstrated strong diagnostic accuracy and has been validated as a reliable predictor of clinical outcomes in various patient populations (18–22). However, studies examining the relationship between the Frailty Index-Lab (FI-Lab) and asthma are limited. The FI-Lab may be particularly suitable for assessing frailty in critically ill patients with asthma.

In this study, we aimed to calculate the FI-Lab score and evaluate its relationship with patient prognosis. We analyzed the clinical data of ICU patients with acute asthma in the Medical Information Mart for Intensive Care (MIMIC-IV) database (2008–2022). We hypothesized that there would be a strong association between the FI-Lab and clinical outcomes in patients with asthma. Furthermore, we hypothesized that patients with severe asthma and higher FI-Lab scores would have longer ICU stays and a higher risk of mortality. We provide new perspectives and tools for the prognostic assessment of critically ill patients with asthma, thereby informing clinical decision-making.

## Methods

### Database and study population

Patients diagnosed with asthma were enrolled using the MIMIC-IV database (version 3.0). This database comprises 546,028 hospital admission records and 94,458 ICU admission records from the Beth Israel Deaconess Medical Center, Boston, Massachusetts, USA, from 2008 to 2022 (23). Permission to access the MIMIC-IV database was granted to Yi Yu, one of the authors (certificate ID: 6477678). The study complied with the Guidelines for Strengthening the Reporting of Observational Studies in Epidemiology (STROBE) (24).

### Inclusion and exclusion criteria

**Figure 1:**
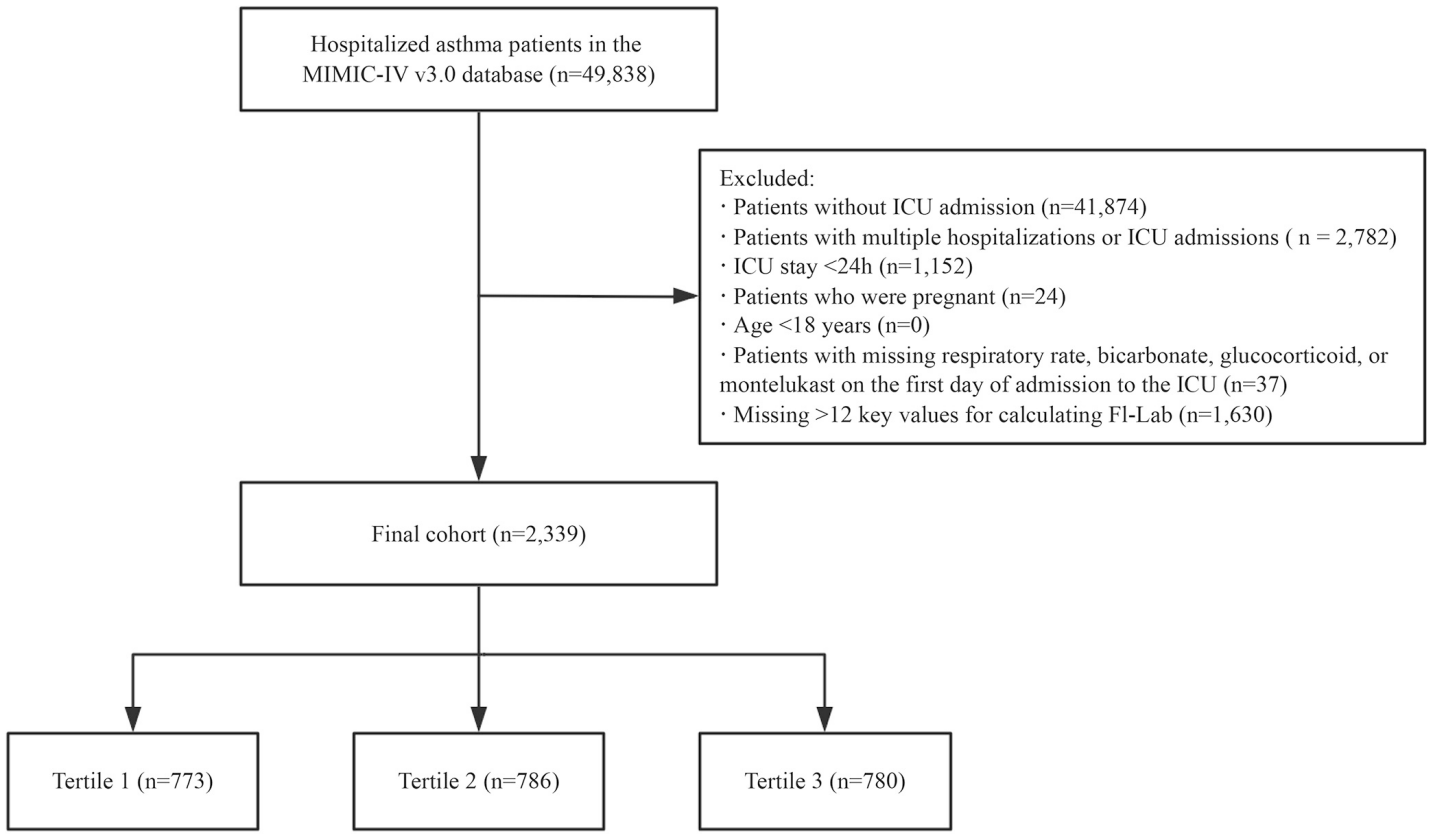
Flowchart outlining the process for enrollment of study participants.

Our study population comprised patients diagnosed with asthma (based on the International Classification of Diseases, Ninth and Tenth Revisions) admitted to the ICU for the first time. Patients were excluded if they met any of the following criteria: (i) age < 18 years, (ii) ICU length of stay < 24 h, (iii) pregnancy, (iv) missing data on respiratory rate, bicarbonate, glucocorticoid, or montelukast on the first day of ICU admission, and (v) insufficient data to construct the FI-Lab scale (missing >12 items; Figure 1).

### Construction of the FI-Lab

The FI-Lab scale was constructed using 33 items, including 30 laboratory test results obtained within 6 h before to 24 h after the first ICU admission. These encompassed blood tests (hemoglobin, white blood cell, and platelet counts; alanine transaminase, alkaline phosphatase, total bilirubin, albumin, lactate dehydrogenase, creatinine, blood urea nitrogen, glucose, sodium, potassium, calcium, phosphorus, fibrinogen, and troponin T levels; plasminogen time; international normalized ratio; and activated partial thromboplastin time), arterial blood gas analyses (pH, partial pressure of oxygen, partial pressure of carbon dioxide, and lactate levels), and urinalysis (leukocyte and erythrocyte counts and the levels of protein, glucose, ketone bodies, and bilirubin). Additionally, three vital signs (systolic blood pressure, diastolic blood pressure, and heart rate) were incorporated into the FI-Lab calculation using values averaged over the initial 24 h of ICU admission.

Each item in the FI-Lab scale was dichotomized according to the standard reference ranges provided in the database; values within the reference interval were assigned a score of 0, while values outside the reference interval were assigned a score of 1. The reference values for each parameter are listed in the Supplementary Table S1. The FI-Lab score was calculated by summing the scores for all items and dividing them by the total number of items, yielding a scale ranging from 0 to 1.

### Covariates

In addition to the items necessary for calculating the FI-Lab, demographic and admission information was collected, including age, sex, race, smoking history, obesity, respiratory rate, bicarbonate, Partial pressure of arterial oxygen/fraction of inspired oxygen (PaO_2_/FiO_2_), length of ICU stay, 28-day, ICU, and 90-day survival status, acute physiology score III (APSIII), Sequential Organ Failure Assessment (SOFA) scores, Acute Physiology and Chronic Health Evaluation II (APACHE II), Charlson comorbidity index (CCI), and comorbidities such as heart failure, hypertension, diabetes, chronic obstructive pulmonary disease (COPD), acute exacerbation of bronchial asthma (AEBA), cardiac shock, and sepsis. Interventions were classified by time of first use: MV and vasoactive therapy if started ≤ 24 h after ICU admission; glucocorticoids (total course) and montelukast (total dose and course) initiated (a) ≤ 24 h after ICU admission, (b) anytime during ICU stay, or (c) prior to ICU admission. Multicollinearity was evaluated by calculating the variance inflation factor (VIF) for the variables included in the analysis. A VIF >2 was considered indicative of multicollinearity.

### Outcome

The primary outcome of interest was 28-day mortality, whereas the secondary outcomes were ICU and 90-day mortality in patients with asthma.

### Statistical analysis

Baseline patient characteristics were stratified into different groups. Categorical variables are expressed as numbers (percentages), whereas continuous variables are reported as mean ± standard deviation or median (interquartile range), depending on their distribution. Differences in continuous variables between groups were analyzed using analysis of variance or rank-sum tests, as appropriate. For categorical variables, chi-square or Fisher's exact tests were employed to compare group characteristics.

The distribution of missing values for all variables is detailed in Supplementary Table S2 of the supplementary material. The proportion of missing data for all covariates was < 1% across all the analyses except for PaO_2_/FiO_2_. We directly excluded variables with missing values < 1%, and then used a multivariate single imputation method based on an iterative imputer, employing a Bayesian Ridge model as the estimator in each step of the round-robin imputation process (25). Restricted cubic spline (RCS) curves were applied to illustrate the relationship between FI-Lab levels and mortality risk. Multivariate Cox regression analysis was conducted to evaluate the correlation between the FI-Lab and mortality. The adjusted Cox model was used to account for various covariates in the model. Three models were employed for the regression analysis. Subgroup and interaction analyses were also conducted to adjust for relevant covariates.

The 28-day mortality rate was assessed using Kaplan–Meier survival analysis, stratified according to FI-Lab groupings, with differences evaluated using the log-rank test. Stratified and interaction analyses were conducted based on key variables, including age (< 60 or ≥60 years), sex (male or female), race (white or non-white), presence of sepsis (yes or no), MV status (yes or no), heart failure (yes or no), and SOFA score (< 5 or ≥5).

ROC curve analysis was conducted to evaluate the discriminative power of the FI-Lab, SOFA, and APACHE II scores for predicting 28-day mortality. The AUC and its 95% CI were calculated for each score. The optimal cutoff was identified using the Youden index, and pairwise comparisons of AUCs were made using DeLong's test.

All statistical analyses were performed using STATA software (version 17.0), R software (http://www.R-project.org, The R Foundation), and Free Statistics software version 1.9.2 (26). A two-tailed *p*-value of < 0.05 was considered statistically significant. Although an *a-priori* sample-size calculation was not feasible, a *post-hoc* power analysis (PASS 2025) based on the observed HR = 1.32, event rate = 13.2%, and covariate *R*^2^ = 0.20 indicated 98% power (two-sided α = 0.05). We note that *post-hoc* power is descriptive and does not replace prospective sample-size planning.

## Results

### Participants

A total of 49,961 patients met the study criteria for asthma. After excluding those with >12 key values missing (required for calculating the FI-Lab), repeated ICU admissions, ICU stays of < 24 h, diagnosis sequence numbers >5, pregnant patients, patients under the age of 18, and those with missing respiratory rate, bicarbonate, glucocorticoid, or montelukast data on the first day of ICU admission, the final cohort consisted of 2,339 patients. The detailed selection process for the study participants is presented in Figure 1.

### Linear relationship between the FI-Lab and mortality

**Figure 2:**
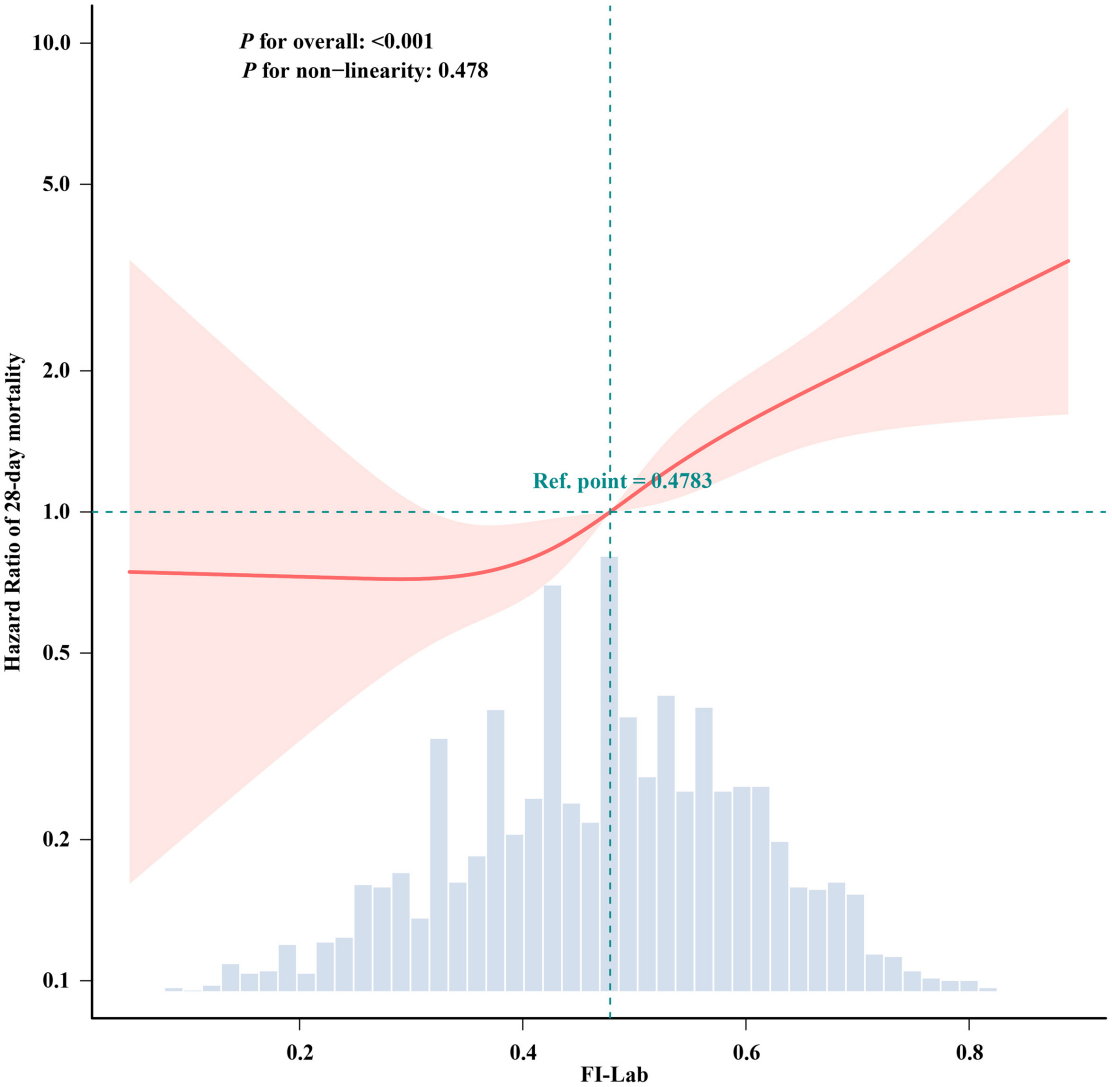
Spline curves showing the association of the FI-Lab as a continuous variable with 28-day mortality. The spline curves were adjusted for all factors of Model 3 in multivariable Cox regression.

The RCS analysis revealed a linear relationship between FI-Lab levels at ICU admission and the risk of 28-day mortality in patients with asthma (*p* for non-linearity: 0.478). An FI-Lab value of 0.48 corresponded to a hazard ratio (HR) of 1.00. Overall, the risk of 28-day mortality increased progressively with increasing FI-Lab levels (Figure 2).

### Baseline characteristics

The study included 2,339 patients with a mean age of 61.3 years, of whom 43.2% were male. Based on the tertiles of the FI-Lab at ICU admission (T1: < 0.43, T2: 0.43–0.54, T3: ≥0.54), the study participants' baseline characteristics were analyzed and presented in Table 1 and Supplementary Table S3. Comparative analysis among the three FI-Lab groups revealed significant differences. Patients in the low FI-Lab group were generally younger and had lower APSIII, CCI, and SOFA scores, lower rates of sepsis, shorter ICU stays, and reduced use of MV and vasopressors than in the other groups.

**Table 1:** Baseline characteristics and outcomes of participants classified by FI-Lab tertiles.

**Variables**	**Total (*n* = 2,339)**	**T1 (FI-Lab < 0.43)**	**T2 (0.43 ≤ FI-Lab < 0.54)**	**T3 (FI-Lab ≥0.54)**	***p*-Value**
		**(*****n*** = **773)**	**(*****n*** = **786)**	**(*****n*** = **780)**	
**Demographics**					
Age, years	61.3 ± 17.1	59.5 ± 18.1	63.1 ± 16.4	61.2 ± 16.7	< 0.001
Male, *n* (%)	1,011 (43.2)	316 (40.9)	367 (46.7)	328 (42.1)	0.049
Race/white, *n* (%)	1,376 (58.8)	442 (57.2)	483 (61.5)	451 (57.8)	0.180
Smoking history, *n* (%)	197 (8.4)	63 (8.2)	72 (9.2)	62 (7.9)	0.652
Obesity, *n* (%)	520 (22.2)	150 (19.4)	179 (22.8)	191 (24.5)	0.050
**Vital signs**					
Respiratory rate, bpm	29.3 ± 6.9	28.3 ± 6.8	29.1 ± 6.6	30.4 ± 7.1	< 0.001
**Laboratory parameters**					
Bicarbonate, mmol/L mmHg	24.1 ± 4.6	24.9 ± 4.1	24.5 ± 4.7	22.9 ± 4.7	< 0.001
PaO_2_/FiO_2_, mmHg	175.0 (110.0, 268.0)	203.7 (129.5, 305.0)	173.2 (116.0, 248.5)	156.3 (95.0, 256.6)	< 0.001
**Clinical severity scores**					
APSIII	47.4 ± 21.9	36.5 ± 14.5	45.1 ± 18.9	60.8 ± 24.0	< 0.001
SOFA	5.0 (2.0, 7.0)	3.0 (1.0, 5.0)	5.0 (3.0, 7.0)	7.0 (4.0, 10.0)	< 0.001
APACHE II	18.4 ± 7.7	14.2 ± 6.3	18.4 ± 6.5	22.6 ± 8.0	< 0.001
CCI	5.0 (3.0, 7.0)	4.0 (2.0, 6.0)	5.0 (3.0, 7.0)	6.0 (4.0, 8.0)	< 0.001
**Intervention**					
MV^a^, *n* (%)	1,206 (51.5)	333 (43.1)	437 (55.6)	436 (55.9)	< 0.001
Vasopressors^a^, *n* (%)	900 (38.5)	185 (23.9)	333 (42.4)	382 (49.0)	< 0.001
Glucocorticoid^a^, *n* (%)	463 (19.8)	152 (19.7)	143 (18.2)	168 (21.5)	0.250
Glucocorticoid^b^, *n* (%)	632 (27.0)	201 (26)	195 (24.8)	236 (30.3)	0.039
Glucocorticoid^c^, *n* (%)	165 (7.1)	45 (5.8)	58 (7.4)	62 (7.9)	0.238
Montelukast^a^, *n* (%)	148 (6.3)	58 (7.5)	50 (6.4)	40 (5.1)	0.157
Montelukast^b^, *n* (%)	184 (7.9)	75 (9.7)	60 (7.6)	49 (6.3)	0.042
Montelukast^c^, *n* (%)	114 (4.9)	37 (4.8)	41 (5.2)	36 (4.6)	0.850
**Comorbidities**					
Heart failure, *n* (%)	654 (28.0)	159 (20.6)	222 (28.2)	273 (35.0)	< 0.001
Hypertension, *n* (%)	1,471 (62.9)	458 (59.2)	523 (66.5)	490 (62.8)	0.012
Diabetes, *n* (%)	679 (29.0)	194 (25.1)	252 (32.1)	233 (29.9)	0.008
COPD, *n* (%)	143 (6.1)	47 (6.1)	48 (6.1)	48 (6.2)	0.998
AEBA, *n* (%)	113 (4.8)	48 (6.2)	38 (4.8)	27 (3.5)	0.041
Cardiac shock, *n* (%)	137 (5.9)	21 (2.7)	36 (4.6)	80 (10.3)	< 0.001
Sepsis, *n* (%)	1,403 (60.0)	325 (42.0)	483 (61.5)	595 (76.3)	< 0.001
**Clinical outcomes**
ICU LOS, days	2.8 (1.7, 5.5)	2.3 (1.5, 4.1)	2.7 (1.5, 5.4)	3.6 (2.1, 7.2)	< 0.001
ICU mortality, *n* (%)	199 (8.5)	13 (1.7)	49 (6.2)	137 (17.6)	< 0.001
28-day mortality, *n* (%)	309 (13.2)	34 (4.4)	84 (10.7)	191 (24.5)	< 0.001
90-day mortality, *n* (%)	397 (17.0)	48 (6.2)	112 (14.2)	237 (30.4)	< 0.001

^a^Within 24 h of ICU admission.

^b^At any time during the ICU stay.

^c^Prior to ICU admission.

FI-Lab, the physiological and laboratory-based frailty index; T, tertiles; PaO_2_/FiO_2_, partial pressure of arterial oxygen/fraction of inspired oxygen; APSIII, acute physiology score III; SOFA, sequential organ failure assessment; APACHE II, Acute Physiology and Chronic Health Evaluation II; CCI, Charlson comorbidity Index; MV, mechanical ventilation; COPD, chronic obstructive pulmonary disease; AEBA, acute exacerbation of bronchial asthma; ICU, intensive care unit; LOS, length of stay.

### Relationship between the FI-Lab and mortality

Univariate analysis revealed a significant association between the FI-Lab and mortality outcomes. A higher FI-Lab was associated with increased 28-day mortality [HR = 1.86, 95% confidence interval (CI): 1.69–2.04, *p* < 0.001], ICU mortality (HR = 1.62, 95% CI: 1.43–1.82, *p* < 0.001), and 90-day mortality (HR = 1.79, 95% CI: 1.65–1.94, *p* < 0.001; Table 2).

**Table 2:** Univariate and multivariate Cox regression models of FI-Lab with mortality in patients with asthma.

**Variable**	**Unadjusted model**		**Model 1**		**Model 2**		**Model 3**	
	**HR 95% CI**	***p*** **-Value**	**HR 95% CI**	***p-*** **Value**	**HR 95% CI**	***p*** **-Value**	**HR 95% CI**	***p*** **-Value**
**28-day mortality**								
FI-Lab (per 0.1 score)	1.86 (1.69–2.04)	< 0.001	1.94 (1.75–2.14)	< 0.001	1.37 (1.22–1.54)	< 0.001	1.32 (1.17–1.50)	< 0.001
**FI-Lab (tertiles)**								
T1 (< 0.43)	1 (ref)		1 (ref)		1 (ref)		1 (ref)	
T2 (0.43–0.54)	2.51 (1.68–3.73)	< 0.001	2.49 (1.67–3.72)	< 0.001	1.71 (1.14–2.56)	0.010	1.65 (1.10–2.50)	0.016
T3 (≥0.54)	6.32 (4.39–9.11)	< 0.001	6.52 (4.53–9.41)	< 0.001	2.57 (1.72–3.84)	< 0.001	2.35 (1.55–3.56)	< 0.001
*p* for trend		< 0.001		< 0.001		< 0.001		< 0.001
**ICU mortality**								
FI-Lab (per 0.1 score)	1.62 (1.43–1.82)	< 0.001	1.68 (1.48–1.91)	< 0.001	1.29 (1.11–1.51)	0.001	1.25 (1.05–1.47)	0.011
**FI-Lab (tertiles)**								
T1 (< 0.43)	1 (ref)		1 (ref)		1 (ref)		1 (ref)	
T2 (0.43–0.54)	3.06 (1.66–5.64)	< 0.001	2.99 (1.62–5.53)	< 0.001	2.17 (1.16–4.04)	0.015	2.05 (1.09–3.87)	0.026
T3 (≥0.54)	5.92 (3.35–10.48)	< 0.001	6.13 (3.46–10.87)	< 0.001	2.83 (1.52–5.27)	0.001	2.35 (1.24–4.44)	0.009
*p* for trend		< 0.001		< 0.001		0.001		0.015
**90-day mortality**								
FI-Lab (per 0.1 score)	1.79 (1.65–1.94)	< 0.001	1.86 (1.71–2.03)	< 0.001	1.37 (1.24–1.52)	< 0.001	1.33 (1.20–1.48)	< 0.001
**FI-Lab (tertiles)**								
T1 (< 0.43)	1 (ref)		1 (ref)		1 (ref)		1 (ref)	
T2 (0.43–0.54)	2.39 (1.71–3.36)	< 0.001	2.37 (1.69–3.32)	< 0.001	1.71 (1.22–2.42)	0.002	1.66 (1.17–2.35)	0.004
T3 (≥0.54)	5.74 (4.21–7.83)	< 0.001	5.92 (4.34–8.08)	< 0.001	2.64 (1.87–3.72)	< 0.001	2.44 (1.72–3.46)	< 0.001
*p* for trend		< 0.001		< 0.001		< 0.001		< 0.001

Model 1: adjusted for age, gender, race, obesity, and smoking history.

Model 2: model 1 + respiratory rate, bicarbonate, PaO_2_/FiO_2_, APSIII, SOFA, APACHE II, CCI.

Model 3: model 2 + adjusted for pre-ICU exposure to glucocorticoids and montelukast, day-1 administration of MV, vasopressors, glucocorticoids and montelukast, cumulative in-ICU use and duration (days) of glucocorticoids, cumulative in-ICU use, duration (days) and total dose (mg) of montelukast, heart failure, hypertension, diabetes, COPD, AEBA, cardiac shock, sepsis.

FI-Lab, the physiological and laboratory-based frailty index; HR, hazard ratio; CI, confidence interval; T, tertiles; PaO_2_/FiO_2_, partial pressure of arterial oxygen/fraction of inspired oxygen; APSIII, acute physiology score III; SOFA, sequential organ failure assessment; APACHE II, Acute Physiology and Chronic Health Evaluation II; CCI, Charlson comorbidity Index; MV, mechanical ventilation; COPD, chronic obstructive pulmonary disease; AEBA, acute exacerbation of bronchial asthma.

Extended multivariate Cox regression analysis (Table 2) consistently demonstrated significant HRs across all models: HRs ranged from 1.32 to 1.94 for 28-day mortality (*p* < 0.001 for all), 1.25 to 1.68 for ICU mortality (*p* < 0.05 for all), and 1.33 to 1.86 for 90-day mortality (*p* < 0.001 for all). After adjusting for all covariates, a 0.1-unit increase in the FI-Lab was associated with a 32% increase in the risk of 28-day mortality (HR = 1.32, 95% CI: 1.17–1.50, *p* < 0.001, Model 3), a 25% increase in ICU mortality risk (HR = 1.25, 95% CI: 1.05–1.47, *p* = 0.011, Model 3), and a 33% increase in 90-day mortality risk (HR = 1.33, 95% CI: 1.20–1.48, *p* < 0.001, Model 3).

### Discriminative performance of FI-Lab

ROC curve analysis indicated an AUC of 0.723 (95% CI 0.692–0.753) for FI-Lab in predicting 28-day mortality, with an optimal cut-off of 0.142 providing a sensitivity of 0.647 and specificity of 0.702 (Supplementary Figure S1). Comparatively, SOFA and APACHE II showed AUCs of 0.712 (95% CI 0.680–0.744) and 0.746 (95% CI 0.718–0.774), respectively. No significant differences in discriminative ability were found when tested against FI-Lab (*p* = 0.443 for SOFA and *p* = 0.146 for APACHE II; Supplementary Figure S2, Supplementary Table S4

### Kaplan–Meier survival curve analysis

**Figure 3:**
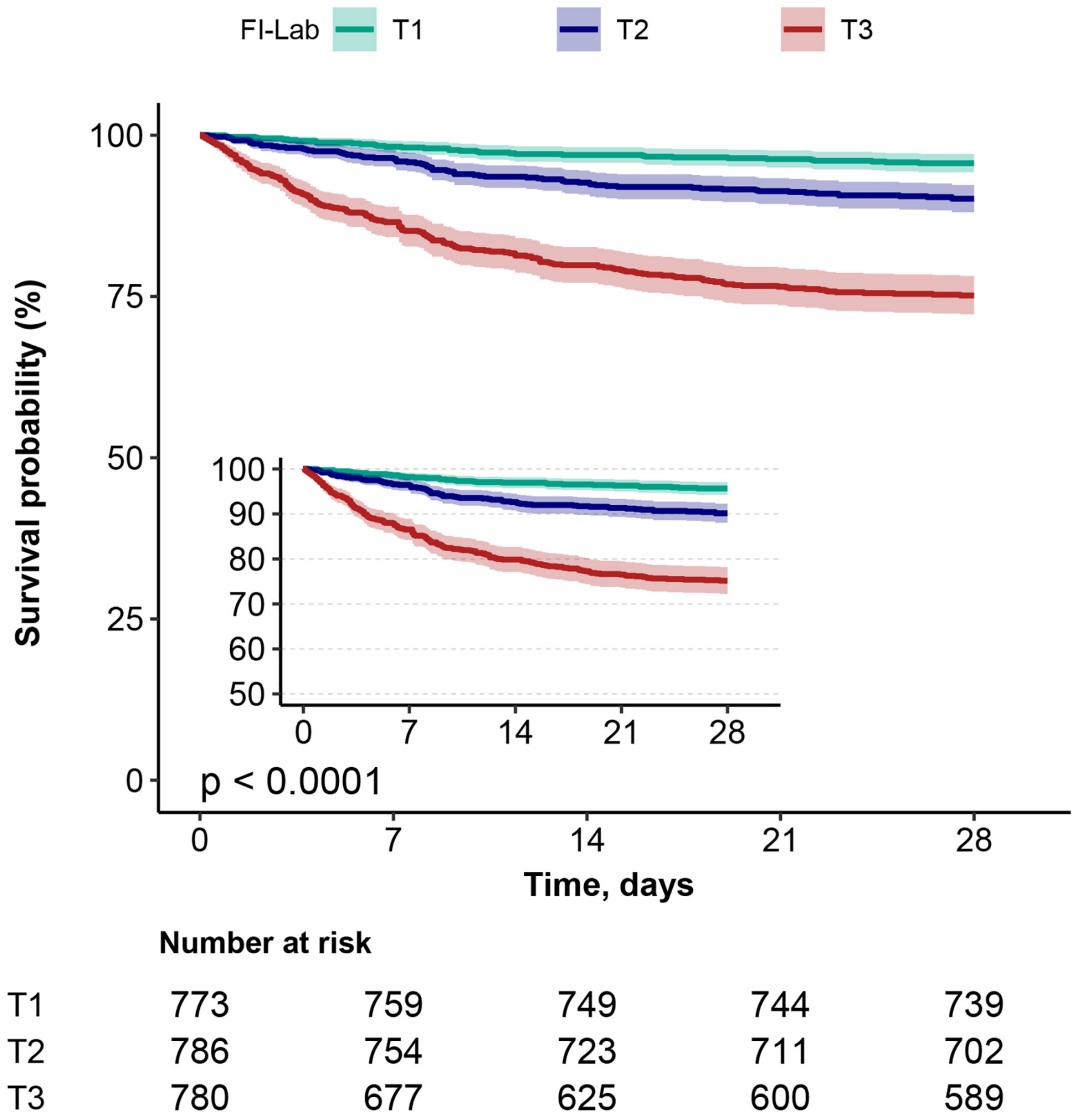
Kaplan–Meier survival curves, categorized by the FI-Lab, for patients with asthma at day 28.

Kaplan–Meier survival curves (Figure 3) grouped by the three FI-Lab categories showed significantly lower cumulative survival times during hospitalization as the FI-Lab increased (log-rank test, *p* < 0.001).

### Subgroup analysis and sensitivity analysis

**Figure 4:**
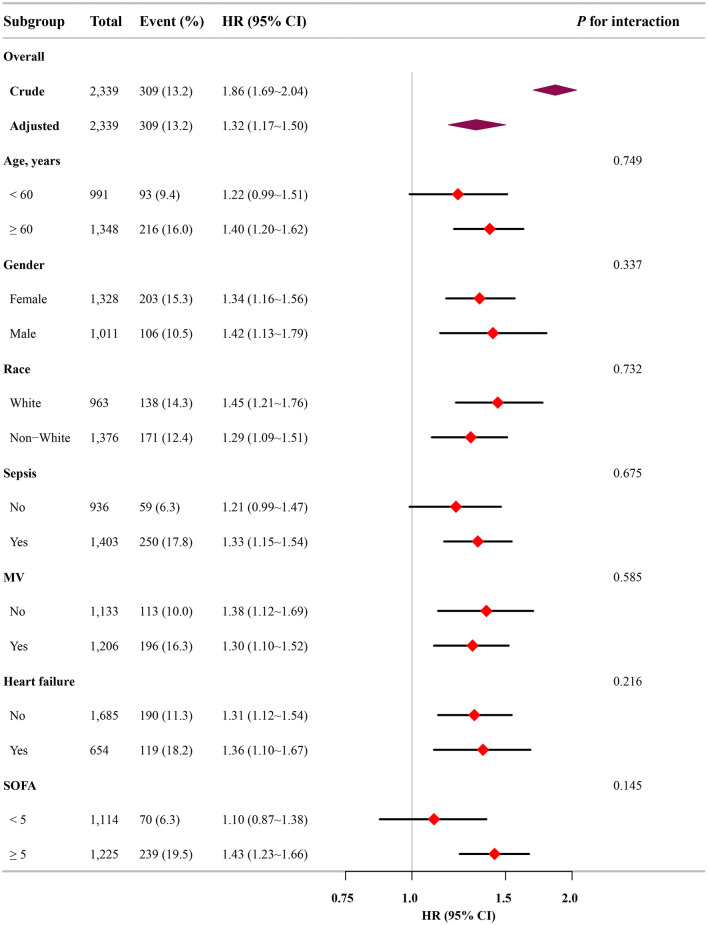
Association between the FI-Lab and patients with asthma at 28-day mortality in relation to the baseline characteristics.

Subgroup analysis further confirmed the robustness and reliability of the association between the FI-Lab and mortality. No significant interactions were observed across the subgroups, indicating consistent effects (*p*-value for interaction >0.05; Figure 4).

When the FI-Lab was analyzed as a categorical variable, consistent and significant HRs were observed across all models for 28-day mortality, with HRs ranging from 1.65 to 6.52 (*p* < 0.05 for all). Similar trends were noted for ICU and 90-day mortality, reinforcing the association between higher FI-Lab levels and increased mortality risk (Table 2).

## Discussion

### Main findings

Our study represents the most comprehensive cohort investigation to date that explores the impact of the FI-Lab score on mortality among ICU patients with asthma. Our results suggest that patients with asthma and a higher FI-Lab score exhibit a significantly increased risk of mortality at 28 days, in the ICU, and at 90 days. Notably, these associations remained robust even after the FI-Lab was treated as a categorical variable. Subgroup analyses and Kaplan–Meier survival curves corroborated these findings, further validating the observed associations. The RCS analysis demonstrated a linear relationship between the FI-Lab and mortality risk, revealing a progressive increase in mortality risk as the FI-Lab increased. Accordingly, an elevated FI-Lab in ICU-admitted patients with asthma may serve as an essential indicator of poor in-hospital prognosis.

### Effects of FI-Lab and mortality for critically-ill patients with asthma

The novel FI-Lab has demonstrated robust prognostic and predictive values in various disease states (18, 19, 21, 22). Recent research has demonstrated that the FI-Lab is a reliable predictor of in-hospital mortality in critically ill ICU patients. Furthermore, combining the FI-Lab with other frailty measures may enhance the identification of critically ill patients at a higher risk of in-hospital mortality (27). Nevertheless, research examining the use of the FI-Lab in ICU patients with asthma is lacking. This study represents an innovative systematic investigation of the application of the FI-Lab in this patient population, and the findings are noteworthy.

Our results are consistent with those of previous studies. Older patients with moderate-to-severe asthma are more prone to frailty, which is often accompanied by significant multisystem functional decline. Patients with higher levels of frailty demonstrate poorer asthma control, as evidenced by an increased symptom burden and frequency of acute exacerbations. Additionally, they exhibit a lower quality of life, as indicated by their limited physical functioning and poor mental health (28, 29). Patients of advanced age with moderate-to-severe asthma who display reduced grip strength are at an elevated risk of developing frailty. Grip strength measurement is an effective method to differentiate between patients with and without frailty. This measurement can be readily employed in clinical practice as a straightforward, non-invasive instrument to promptly identify frailty risk (30). A correlation has been identified between the incidence of myasthenia gravis and the presence of underlying asthma in older individuals (31). However, patients in the ICU are frequently administered analgesics and sedatives, which presents a significant challenge to the implementation of this frailty scoring method.

A chronic inflammatory state associated with asthma may precipitate a decline in functional capacity and increase the risk of frailty. Conversely, the presence of frailty may also increase the risk of developing asthma owing to the impact of frailty on respiratory function and immune responses (14, 32). The findings of this study indicate a significant association between the FI-Lab and mortality in ICU patients with asthma. The FI-Lab can be employed as a risk stratification instrument for the prognostic assessment of patients with asthma, providing theoretical support for its application.

### Implications for clinical practice

It is vital to identify patients in the ICU who are frail to implement the appropriate management strategies. For example, this can be achieved by encouraging physical activity and providing nutritional supplements. This approach may improve patient prognosis (33–35). Additionally, the early identification of frailty in the ICU can lead to tailored and effective interventions, reducing mortality and improving long-term outcomes.

The FI-Lab score provides an objective measure of frailty, supporting data-driven decision-making and enhancing the quality of care. It can also facilitate discussions with patients and families, helping to set realistic expectations. However, the FI-Lab score is intended to optimize care, not deny treatment. It should be used alongside clinical judgment to ensure all patients receive appropriate care.

### Strengths of our study

Our study has several notable strengths. First, we used a comprehensive and publicly accessible database to ensure the reliability and breadth of the data. Second, to our knowledge, this is the first study to specifically examine the impact of the FI-Lab on mortality risk among ICU patients with asthma. Our findings demonstrate a strong correlation between elevated FI-Lab levels and poor prognosis in this patient population. Third, we employed multiple regression analyses to confirm the robustness and reliability of our results. This rigorous analytical approach strengthens the credibility and internal validity of our findings. Lastly, because FI-Lab is derived solely from readily available laboratory and vital-sign data, it offers discrimination comparable to APACHE II or SOFA yet can be calculated instantly at ICU admission without additional clinical assessments. Clinicians can therefore use FI-Lab to rapidly identify high-risk patients with severe asthma and initiate intensified monitoring and organ-support measures early.

### Limitations of our study

While this study represents the most comprehensive investigation to date on the application of the FI-Lab in ICU patients with asthma, it has several limitations. First, we calculated the FI-Lab only at the time of ICU admission, which does not account for potential changes in FI-Lab values during hospitalization. These dynamic trends, which can be extracted and calculated using an applet, may offer additional prognostic insights. However, the relationship between these changes and the outcomes in ICU patients with asthma remains unclear and requires further exploration. Second, while our findings provide important insights, the single-center nature of the MIMIC-IV database may limit generalizability to other settings. External validation in multicenter cohorts would strengthen the clinical applicability of our results. Multicenter validation is required before wider clinical application. Third, certain factors that may influence asthma mortality, such as pre-hospital care details and functional status, were not reported in the dataset; their absence precluded a more comprehensive analysis of potential contributors to patient outcomes. Fourth, as a retrospective observational study, our design limits causal inference due to potential unmeasured confounders and selection bias; a future RCT is warranted to validate FI-Lab's prognostic role. Finally, several FI-Lab components—albumin, glucose, hemoglobin—are potentially modifiable. Our findings describe an observational association only; whether correcting these markers improves survival remains untested. Following the target-trial emulation framework recently detailed by Yang et al. (36), future studies could apply inverse-probability weighting or doubly robust estimators to quantify the causal effect of interventions directed at modifiable FI-Lab variables and extend follow-up beyond 90 days to determine whether any benefits are sustained.

## Conclusions

This study establishes that elevated FI-Lab levels are strongly associated with poor prognosis in ICU patients with asthma. Clinicians should pay particular attention to patients with asthma who present with high FI-Lab levels upon ICU admission. The FI-Lab offers a novel evidence-based approach for assessing the prognosis of patients with severe asthma and potentially improving clinical outcomes. However, further RCTs are essential to validate the findings and hypotheses presented.

## Data Availability

Publicly available datasets were analyzed in this study. This data can be found here: https://mimic.mit.edu/.
