# Supplementary material for: Association between a frailty index derived from laboratory tests and clinical outcomes in critical care patients with asthma: a retrospective study based on the MIMIC-IV database
Source: Front Med (Lausanne). 2025 Sep 18;12:1539531. doi: 10.3389/fmed.2025.1539531 (PMC12488622; doi:10.3389/fmed.2025.1539531)
Supplement: Supplementary file 2 [file Table_2.docx]

**Table S2.** Missing number (%) for risk variables and outcome variables

| **Risk variable** | **Missing number (%)** |
| --- | --- |
| Age | 0 (0) |
| Gender | 0 (0) |
| Race | 0 (0) |
| Smoking history | 0 (0) |
| Obesity | 0 (0) |
| Respiratory rate | 10 (0.43%) |
| Bicarbonate | 7 (0.30%) |
| APSIII | 0 (0) |
| PaO_2_/FiO_2_ | 979 (41.85%) |
| SOFA | 0 (0) |
| CCI | 0 (0) |
| APACHE II | 0 (0) |
| MV | 0 (0) |
| Vasopressors | 0 (0) |
| Glucocorticoid | 10 (0.43%) |
| Montelukast | 10 (0.43%) |
| Heart failure | 0 (0) |
| Hypertension | 0 (0) |
| Diabetes | 0 (0) |
| COPD | 0 (0) |
| Cardiac shock | 0 (0) |
| AEBA | 0 (0) |
| Sepsis | 0 (0) |
| LOS ICU | 0 (0) |
| ICU mortality | 0 (0) |
| 28-day mortality | 0 (0) |
| 90-day mortality | 0 (0) |

**Abbreviations:** FI-Lab, the physiological and laboratory-based frailty index; APSIII, acute physiology score III; PaO2/FiO2, partial pressure of arterial oxygen/fraction of inspired oxygen; SOFA, sequential organ failure assessment; CCI, Charlson comorbidity Index; APACHE II, Acute Physiology and Chronic Health Evaluation II; MV, mechanical ventilation; COPD, chronic obstructive pulmonary disease; AEBA, acute exacerbation of bronchial asthma; LOS, length of stay; ICU, intensive care unit.
