# Supplementary material for: Association between a frailty index derived from laboratory tests and clinical outcomes in critical care patients with asthma: a retrospective study based on the MIMIC-IV database
Source: Front Med (Lausanne). 2025 Sep 18;12:1539531. doi: 10.3389/fmed.2025.1539531 (PMC12488622; doi:10.3389/fmed.2025.1539531)
Supplement: Supplementary file 3 [file Table_3.docx]

**Table S3.** Montelukast dose/duration and glucocorticoid duration across FI-Lab tertiles

| **Variables** | **Total** | **T1 (FI-Lab<0.43)** | **T2 (0.43≤FI-Lab<0.54)** | **T3 (FI-Lab≥0.54)** | ***P* value** |
| --- | --- | --- | --- | --- | --- |
|  | (n=272) | (n = 99) | (n = 92) | (n = 81) |  |
| Montelukast duration, days | 7.0 (4.0, 12.0) | 5.0 (3.0, 8.0) | 7.0 (4.0, 13.0) | 8.0 (5.0, 16.0) | < 0.001 |
| Montelukast total dosage, mg | 70.0 (40.0, 122.5) | 50.0 (30.0, 80.0) | 80.0 (50.0, 130.0) | 90.0 (50.0, 170.0) | < 0.001 |
|  | (n = 716) | (n = 237) | (n = 220) | (n = 259) |  |
| Glucocorticoid duration, days | 3.0 (1.0, 7.0) | 3.0 (1.0, 6.0) | 4.0 (1.0, 8.0) | 3.0 (1.0, 7.0) | 0.010 |

***Abbreviations***: FI-Lab, the physiological and laboratory-based frailty index; T, tertiles.
