# Supplementary material for: Association between a frailty index derived from laboratory tests and clinical outcomes in critical care patients with asthma: a retrospective study based on the MIMIC-IV database
Source: Front Med (Lausanne). 2025 Sep 18;12:1539531. doi: 10.3389/fmed.2025.1539531 (PMC12488622; doi:10.3389/fmed.2025.1539531)
Supplement: Supplementary file 4 [file Table_4.docx]

**Table S4.** Comparison of prognostic accuracy among FI-Lab, SOFA, and APACHE-II for 28-day mortality

| **Variables** | **AUC(%) (95% CI)** | ***P* value** |
| --- | --- | --- |
| FI-Lab | 72.4% (69.3% ~ 75.4%) |  |
| SOFA | 71.2% (68.0% ~ 74.4%) | 0.443^*^ |
| APACHE II | 74.6% (71.8% ~ 77.4%) | 0.146^＃^ |

***Note***: ^*^ SOFA vs FI-Lab；^#^ APACHE II vs FI-Lab.

***Abbreviations:*** AUC, FI-Lab, the physiological and laboratory-based frailty index; SOFA, sequential organ failure assessment; APACHE II, Acute Physiology and Chronic Health Evaluation II.
