# Supplementary figures and images for: Association between a frailty index derived from laboratory tests and clinical outcomes in critical care patients with asthma: a retrospective study based on the MIMIC-IV database
Source: Front Med (Lausanne). 2025 Sep 18;12:1539531. doi: 10.3389/fmed.2025.1539531 (PMC12488622; doi:10.3389/fmed.2025.1539531)

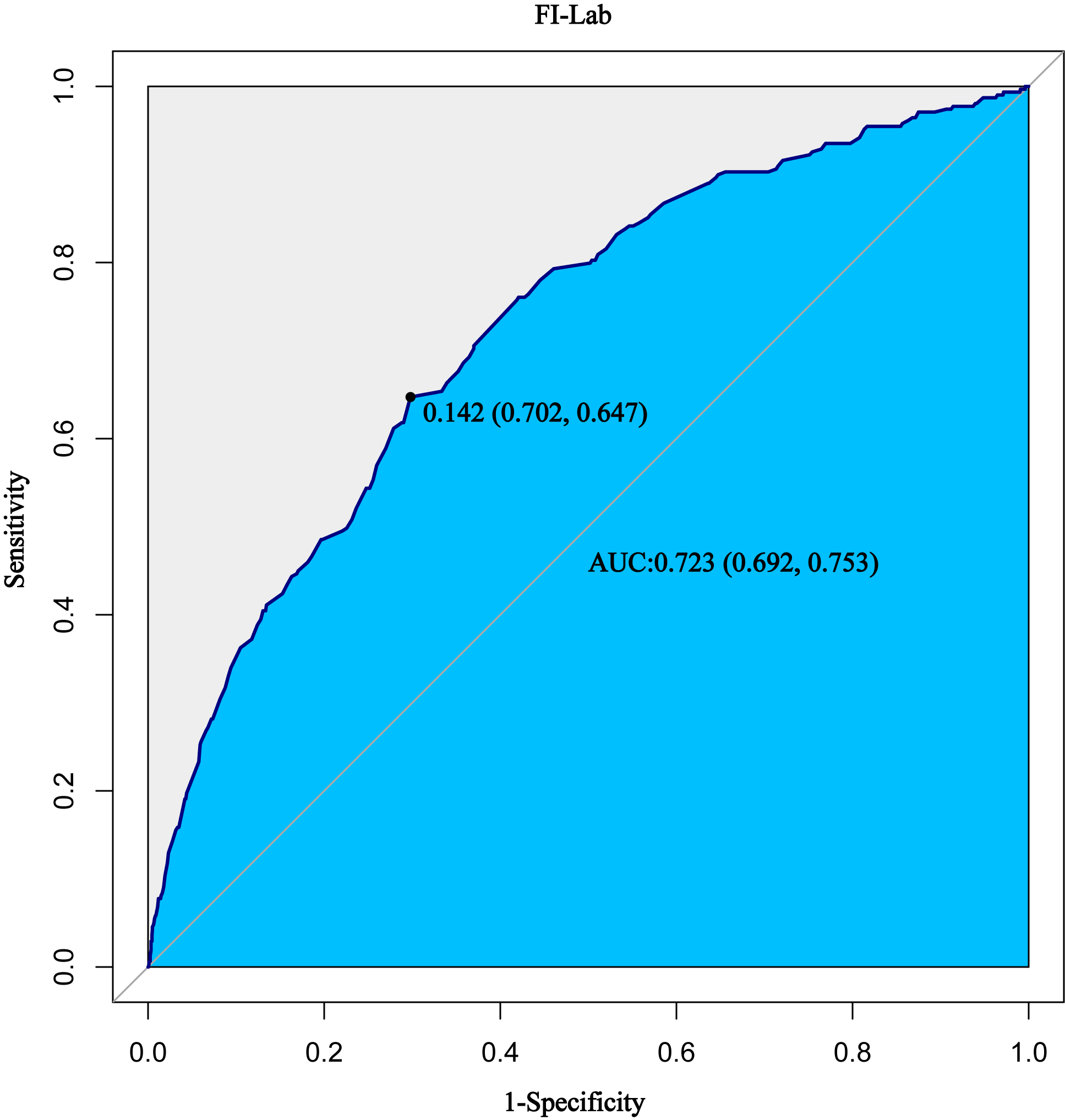

Supplement: Supplementary file 5 [file Image_1.tif]

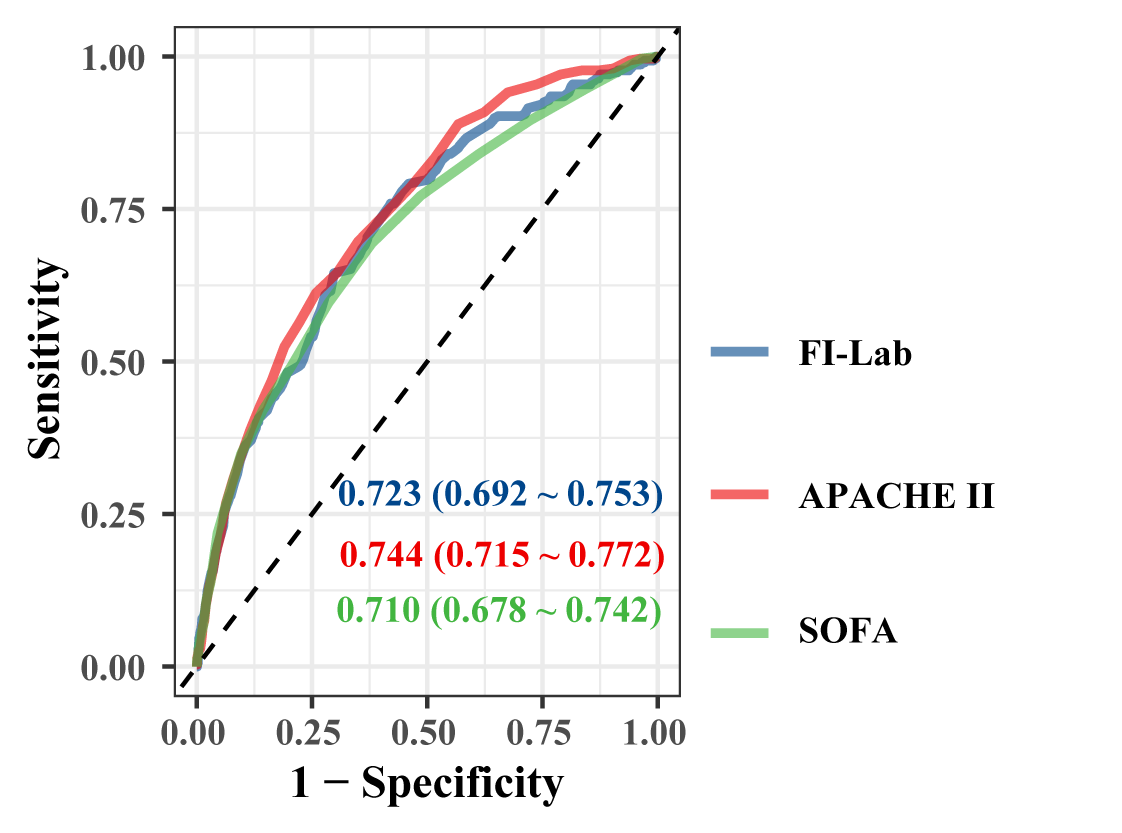

Supplement: Supplementary file 6 [file Image_2.tif]
